# Exploitation of *Aspergillus terreus* for the Production of Natural Statins

**DOI:** 10.3390/jof2020013

**Published:** 2016-04-30

**Authors:** Mishal Subhan, Rani Faryal, Ian Macreadie

**Affiliations:** 1 Department of Microbiology, Faculty of Biological Sciences, Quaid-i-Azam University, Islamabad 45320, Pakistan; mishalsubhan@gmail.com (M.S.); ranifaryal@gmail.com (R.F.); 2 School of Science, RMIT University, Bundoora, Victoria 3083, Australia

**Keywords:** *Aspergillus terreus*, compactin, fermentation, industrial microbiology, lovastatin, mevastatin, mutagenesis, optimization, polyketide

## Abstract

The fungus *Aspergillus (A.) terreus* has dominated the biological production of the “blockbuster” drugs known as statins. The statins are a class of drugs that inhibit HMG-CoA reductase and lead to lower cholesterol production. The statins were initially discovered in fungi and for many years fungi were the sole source for the statins. At present, novel chemically synthesised statins are produced as inspired by the naturally occurring statin molecules. The isolation of the natural statins, compactin, mevastatin and lovastatin from *A. terreus* represents one of the great achievements of industrial microbiology. Here we review the discovery of statins, along with strategies that have been applied to scale up their production by *A. terreus* strains. The strategies encompass many of the techniques available in industrial microbiology and include the optimization of media and fermentation conditions, the improvement of strains through classical mutagenesis, induced genetic manipulation and the use of statistical design.

## 1. Introduction

Statins are polyketide compounds that are produced by some fungi during their secondary metabolism [1]. The statins act as competitive inhibitors, specifically inhibiting HMG-CoA reductase, a rate limiting step of cholesterol biosynthesis. Statins block the conversion of HMG-CoA to mevalonic acid in the mevalonate pathway [1,2,3]. In this metabolic pathway, mevalonate is converted into a number of hydrophobic molecules, sterol isoprenoids and nonsterol isoprenoids [4]. The statins reduce total cholesterol level in serum, especially the low-density lipoprotein levels and are therefore used to treat hypercholesterolemia [5,6].

The statins are the largest selling class of drugs throughout the world. Sales for statins in 2005 were $25 billion [6,7]. In addition to the ability to reduce the risk of cardiovascular morbidity and mortality, statins can also prevent and reduce the development of peripheral vascular disease [8]. Statins not only reduce the LDL-cholesterol levels but also protect against atherosclerotic plaque growth via their antithrombotic and anti-inflammatory effects [9,10,11]. Statins may further be used in cases of hypertension, osteoporotic fractures, ventricular arrhythmia and prevention of Alzheimer’s disease and Parkinson’s disease [12,13,14,15].

## 2. Discovery of Statins

The Japanese microbiologist Dr. Akira Endo pioneered the discovery of statins from the filamentous fungi *Penicillium (P.) citrinum* and later from *A. terreus* in the 1970s. Among the statins, mevastatin was the first to be investigated as a fungal secondary metabolite, later followed by lovastatin (monacolin K or mevinolin) [16,17]. In 1976, Sankyo and Merck & Co commenced collaborative research on statins. Lovastatin was the first statin approved by United States Food and Drug Administration as a hypercholesterolemic drug in August 1987 [18,19]. Many fungi such as *Monascus (M.) ruber* [20], *M. purpureus* [21,22], *M. pilosus, A. terreus* [23,24], *A. flavipes* [25], *A. fischeri, A. flavus, A. umbrosus, A. parasiticus, Accremonium chrysogenum, P. funiculosum, Trichoderma (T.) viridae and T. longibrachiatum* [26] have been reported to produce lovastatin. Lovastatin is the statin of overwhelming interest because it can be produced naturally and its levels can be scaled up using cheap raw materials, reducing the cost of its production in comparison to chemically synthesized statins. According to data from IMS Health, over 10 billion tablets have been distributed and more than 100 million prescriptions have been written worldwide for lovastatin during the years 1988 to 2003. Lovastatin has been marketed for over 20 years with more than 27 million patient-years of therapy [27].

Statins are divided into three different classes depending upon whether their synthesis is natural, semi-synthetic or totally synthetic [19]. Natural statins are produced by direct fermentation of fungi and include lovastatin and pravastatin. Semisynthetic statins include simvastatin that is produced by direct alkylation of lovastatin. The methylbutyrate side chain is converted into a dimethylbutyrate moiety [5,28]. Synthetic statins are different in structure from natural statins, but there is similarity to natural statins in the HMG CoA-like inhibitory moiety. Chemically synthesized statins include atorvastatin, rosuvastatin, fluvastatin and cerivastatin [2,19].

**Figure 1:**
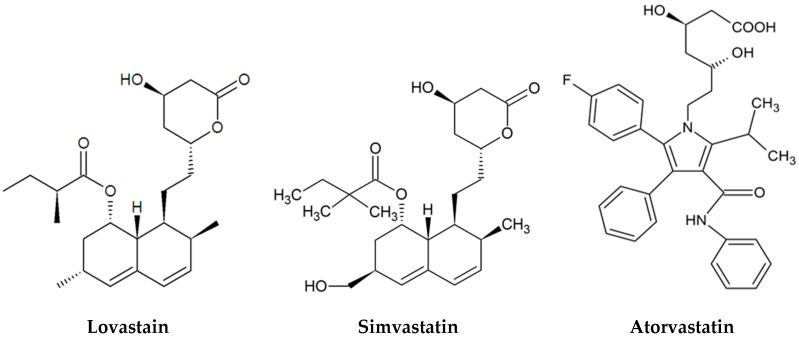
Structure of a natural, semi-synthetic and totally synthetic statin.

Lovastatin and simvastatin are inactive until the uppermost ring is opened by liver enzymes, or by treatment with ethanolic NaOH. Atorvastatin, on the other hand, is produced in active form, with the ring opened (see Figure 1 for examples of the structure of statins). Activated statins have been shown to inhibit the growth of numerous fungi, including *Saccharomyces cerevisiae*, *Candida* spp., *Aspergillus* spp. and *Cryptococcus* spp. through inhibition of HMG-CoA reductase which depletes ergosterol [29,30,31,32,33]. Ergosterol is the fungal equivalent of cholesterol. Both ergosterol and cholesterol are essential for cell viability and preservation of membrane fluidity and both are products of a very similar pathway. 

Statins have potential as antifungals; however, they are unlikely to compete with azoles and drugs that can efficiently and selectively target ergosterol and its biosynthesis in invasive fungal infections. The maximum plasma concentration of the drug is 10–40 ng/mL that can be achieved in clinical trials [34]. Several studies reported the higher MIC values of statins against fungal species ranging from 4 µg/mL to a value greater than 256 µg/mL. The MIC values for the growth inhibition of *A.* *fumigatus* was found to be 58 µg/mL (10 µM) and 0.4 µg/mL (1 µM) in case of atorvastatin and simvastatin, respectively [32]. Lovastatin and simvastatin have the ability to inhibit growth of *Aspergillus* spp. but at concentrations which are toxic to human cells [30,35]. At clinically achievable concentrations, the statins show no effect on the *in vitro* activities of azoles and amphotericin B against the *Aspergillus* spp. [30]. The antifungal activity of fluconazole was studied in combination with lovastatin against *Candida (C.) albicans*. Higher values of MICs were observed with lovastatin in comparison to fluconazole. However, the MIC for fluconazole further decreased when amount of lovastatin was increased in synergy against *C. albicans* [36]. Synergistic antifungal effects of statins and azoles were studied against *Saccharomyces cerevisiae* ATCC 32051 and *C. utilis* Pr_1–2_. Statins increased the activity of azoles at their sub-inhibitory concentrations (SICs) against the yeast strains. Thus the co-administration of these antifungals can increase the potency and reduce the required doses of azoles for fungal treatments, especially in cases of refractory infections [37].

It is also noteworthy that ring-opened forms of lovastatin and simvastatin inhibit the growth of *A. terreus* [30], though the production of lovastatin does not pose a problem to *A. terreus* since it is released outside the cell as the active beta hydroxyl form, possibly as a defense mechanism during a secondary metabolism [38]. Bioassays have been performed for the qualitative screening of lovastatin producers. As lovastatin has the ability to act as an antifungal agent, yeast growth inhibition bioassays [39] and agar well diffusion bioassays using *Neurospora crassa* MTCC-790 as a test strain have been studied [40]. Crude extracts from fungal isolates are loaded into the agar wells and ethyl acetate is used as a control. Strains showing a clear zone of inhibition are selected [39,40].

The active form of lovastatin is converted into the inactive lactone form by the solvent extraction of fermentation media which is less lipophilic compared to the hydroxyl form [41,42]. Lovastatin also has the ability to transform further into methyl ester if methanol is used as a solvent [43,44]. That creates a problem in screening for a high level production since bioassays become more complicated due to the existence of different chemical forms. Thus, there is reliance upon more laborious chemical screening to determine levels of lovastatin in cultures, extracts and fractions [39,45].

## 3. Exploitation of *A. terreus* for Statin Production

Filamentous fungi have the ability to produce secondary metabolites with complex chemical structures. The discovery of new bioactive secondary metabolites and their upscale production is always an aim of both pharmaceutical and agrochemical industries. Fungi are well-established sources for such substances and are exploited to produce a large number of valuable compounds.

*A. terreus* is a filamentous ascomycota, a soil fungus that was originally discovered as a potent producer of lovastatin in 1979. This is the only fungal isolate that has been utilized and commercialized to produce lovastatin [46,47]. Biosynthesis of lovastatin depends not only on composition of culture media like carbon and nitrogen sources, but also on the strain used and culture conditions [48].

Lovastatin production by *A. terreus* can be increased significantly by controlling the culture conditions. Lovastatin production by *A. terreus* is favored by sub-optimal growth conditions [49]. There are also several other environmental factors that influence the production of lovastatin, such as agitation, temperature, pH and moisture content. Agitation interacts with the culturing environments, which in turn affects product formation [45,50,51]. More agitation results in the decrease of dissolved oxygen and increases the shear stress during shake flask fermentation. Low supplementation of dissolved O_2_ (DO) inhibit the product formation [49,51]. An optimum size of inoculum can increase the levels of lovastatin. Large- and small-sized inocula have been reported to reduce the levels of lovastatin [52]. Different sizes of inocula with spore counts ranging from 10^7^ to 10^8^ spores/mL were studied. A spore count of 5 × 10^7^ spores/mL was found to be optimum for the maximum production of lovastatin. Low and high levels of spore count decreased the lovastatin production. A further increase in inoculum size did not increase the amount of lovastatin [52]. High moisture content also decreases the level of lovastatin production due to decreased oxygen availability caused by excessive replacement of air by water, while low levels of moisture content result in failure to reduce metabolic heat during the fermentation process [53]. Optimisation of the pH can positively affect the production of lovastatin during fermentation. The levels of lovastatin were increased at pH range of 7–8.5 but a further increase in pH reduced the productivity [54,55,56]. Last but not the least, temperature is considered as the most important factor influencing the productivity involving the activation and induction of the enzyme required for lovastatin biosynthesis [49]. Different temperatures ranging from 25 to 30 °C were studied. The maximum production of lovastatin was achieved at 30 °C which was found to be the optimum temperature [54]. Cultivation at optimum temperature results in high yields of lovastatin [54,57,58].

### 3.1. Effect of Nutrients on Production of Statins

*A. terreus* has been reported to produce lovastatin in submerged (SmF) batch and fed-batch fermentation along with the solid state fermentation (SSF). A summary of yields and conditions for production of statins in SSF and SmF are shown in Table 1 and Table 2, respectively. 

Gulyamova *et al.* (2013) [36] reported the production of lovastatin by two strains of *Aspergillus terreus*: *A. terreus* 4 and *A. terreus* 20. In both SmF and SSF, five different carbon sources were tested, with the highest yields of lovastatin obtained using lactose as a carbon source. Wheat bran and oat bran were optimised to be the best solid substrate for SSF [61].

Carbon and nitrogen both affect the production of lovastatin from *A. terreus*. According to several studies it has been suggested that high yields can be achieved if nitrogen is the limiting factor. *A. terreus* has the ability to metabolize different kinds of organic and inorganic-defined nitrogen sources. Among them glutamate- and histidine-supplemented media have been reported to enable increased lovastatin production [24,48]. *A. terreus* ATCC 20542 was used for the biosynthesis of lovastatin in optimised culture conditions in SmF, resulting in a three-fold increase in lovastatin levels [47]. *A. terreus* DRCC 122 was used for the production of lovastatin in batch and fed-batch fermentations using corn steep liquor and maltodextrin as nitrogen and carbon sources, respectively, increasing the levels of lovastatin [75]. Fermentation of *A. terreus MIM* A1 and A2 strains on soybean flour and glycerol has been reported to produce lovastatin, mevastatin, pravastatin and monacolin J. 83% of lovastatin was associated with the mycelium and 17% was free in the culture filtrate [76].

*A. terreus* has also been reported to accumulate simvastatin, derived from lovastatin, as a final product of fermentation [77]. Gulyamova *et al.* (2014) described the composition of statins produced by indigenous strain of *A. terreus* 20 in SmF. Statins were extracted from the biomass with acetonitrile after centrifugation and samples were dried for analysis by LC-MS-MS. Lovastatin was detected in lactone, acidic and methyl ester forms. In addition to lovastatin, monacolin L, simvastatin and pravastatin were also detected [77].

Nutritional parameters for increased yields of simvastatin by *A. terreus* have also been reported. An increase of the carbon/nitrogen ratio led to an elevated simvastatin titre in chemically defined media [73]. This agrees with the nitrogen limitation results described above.

### 3.2. Feedback Inhibition Regulation Strategy

Product inhibition as a result of fermentation is a key element to be kept in mind during industrial scale production. The biosynthesis of lovastatin by *A. terreus* involves feedback inhibition. Suppressing this mechanism can greatly enhance the production of lovastatin in fermentation media; however, the exact process is still unknown [47,66,70].

### 3.3. Effect of Other Additives 

Secondary metabolism is usually triggered when primary metabolism is inhibited. Various additives have been placed into culture to improve production of lovastatin. The effects of these additives on lovastatin yields are summarised in Table 3.

The biosynthesis of lovastatin by *A. terreus* is always accompanied by the production of various kinds of intermediate metabolites, especially acids such as itaconic acid, citric acid, pyruvic acid and acetic acid. These acids can easily accumulate in the media and reduce the pH and thereby decrease the amount of lovastatin produced. Low pH affects the formation of enzymes required for the synthesis of lovastatin, so to overcome this effect different kinds of additives have been used [47,80].

Addition of different kinds of polyketide antibiotics resulted in increased production of lovastatin by inhibiting intermediary compounds [66]. Lovastatin production was further increased by 9.2% if 0.5 g/L itaconic acid was added to fermentation medium of *A. terreus* ATCC 20542, resulting in feedback inhibition of undesired metabolites [47].

Patil *et al.* (2011) reported the effect of carboxy methyl cellulose (CMC) on production of lovastatin by *A. terreus* PM3. The presence of CMC restricted the filamentous growth and resulted in pellet formation, stimulating lovastatin production [62]. The impact of exogenous cell signalling molecules has also been studied. The production of lovastatin was increased 1.8-fold upon addition of linoleic acid, the precursor of oxylipin, from *A. terreus* during batch fermentation. Oxylipins are linoleic acid-derived quorum sensing signalling molecules [65]. In recent studies butyrolactone I was added in the bioreactor, resulting in a 2.5-fold increase in both lovastatin and its own production. Butyrolactone I is also a quorum-sensing molecule in *A. terreus*. The study suggested that butyrolactone I is a growth phase-specific inducer for the lovastatin and an auto-stimulator on its own production [78]. The effect of B-group vitamins on the biosynthesis of lovastatin by *A. terreus* ATCC 20542 was also reported. Supplementation of fermentation media with nicotinamide, pyridoxine and calcium D-pantothenate, separately and in mixtures increased the volumetric and specific production of lovastatin [79]. Metal ions, including Zn^2+^, Fe^2+^, Mg^2+,^ Ca^2+^, Cu^2+^ and Mn^2+^ can also alter the cell biochemistry if their concentrations are too high or too low. They can affect cell growth and metabolite production. Among the metal ions, Zn^2+^ and Fe^2+^ at concentration of 5 mM enabled the highest production of lovastatin, 523.9 ± 14.9 mg/L and 406.0 ± 7.8 mg/L respectively [67].

### 3.4. Mutagenesis for Strain Improvement

Wild-type *A. terreus* strains isolated from natural environments usually produce very low levels of statins. Various kinds of strain improvement techniques have been applied to achieve high titres of statins. Strain improvement not only increase the yields of desired metabolites but also removes the unwanted co-metabolites, improves downstream processing by the alteration cellular morphology facilitating the oxygen transfer and improving the utilization of raw sources of carbon and nitrogen [81].

Most of the methods used for the hyper-production of statins in *A. terreus* species include:

- Chemical mutagenesis, involving use of mutagenic chemicals such as ethyl methanesulfonate (EMS) and *N*-methyl-*N*’-nitro-*N*-nitrosoguanidine (NTG) [45,82,83] and
- Physical mutagenesis, involving the use of radiation such as high radiation heavy ion beams and ultraviolet radiation [57,71].

The improved yields following mutagenesis are listed in Table 4.

### 3.5. Systems Biology and Application of A. terreus Genome Knowledge

**Figure 2:**
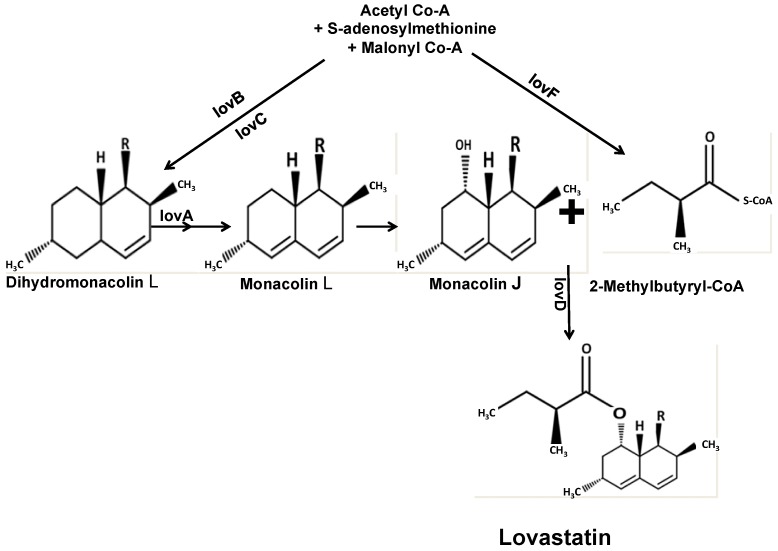
Pathway leading to lovastatin biosynthesis.

Genomic studies on *Aspergillus* spp. reveal that *A. terreus* is unique in possessing gene clusters involved in the biosynthesis of lovastatin [87,88]. The pathway for lovastatin biosynthesis, shown in Figure 2, involves the joining of two polyketides by a polyketide synthase system (PKS). This PKS further comprises two domains, the lovastatin nonaketide synthase (LNKS) and lovastatin diketide synthase (LDKS) [89,90,91,92].

Systems biology approaches have allowed *A. terreus* MF22 to be genetically engineered to achieve a nine-fold increase in lovastatin levels in fermentation broth [93]. Transcriptional profiles were generated after the construction of genomic fragment microarrays from genome of respective strain. Metabolite identification and profiling were done using HPLC-electrospray MS, quadrupole–time of flight MS (TOF-MS) and NMR [93]. We consider that efforts involving systems biology have been very few to date and that further genetic engineering could be performed to further increase the levels of statin production.

### 3.6. Statistical Designing

Different kinds of statistical design models have been applied for the efficient and economic production of statins by *A. terreus*. These designs are used as tools to select the key factors from a multivariable system. Optimization of different parameters in a fermentation system leads to enhanced production of desired compounds minimizing the error in that system. A one-factor-at-a-time (OFAT) approach has been considered to be a conventional and time-consuming technique for optimization of culture media [94,95]. Most of the methods that have been used so far include:

- Response surface methodology (RSM)
- Central composite design (CCD)
- Box−Behnken design (BBD)
- Plackett−Burman (PB)
- Taguchi design

A summary of yields after applying new methods based on models are outlined in Table 5.

Response surface methodology includes the set of mathematical and statistical calculations useful for experimental designing of factors required for desirable responses and optimum conditions with the least experimental trials [95,99,100]. Central composite design results in gathering a large amount of information with a very limited number of experimental trials [72]. Box−Behnken design is an optimization tool for the calculation of responses at intermediate levels of an experiment [101]. Yields of the statins can be increased by inoculating older spores. Spore age was determined using modified Box–Behnken design. The final yields of statins increased to 52% [23]. Goswami *et al.* (2013) used Taguchi design for the optimization of media during production of lovastatin by *A. terreus* JX081272. Signal-to-noise ratio was used to determine the optimum levels and interaction effects [98]. Syed and Rajasimman (2015) reported the enhanced production of mevastatin by applying Plackett–Burman and central composite design [59]. Improved production of lovastatin was observed with an increase of 2.6-fold under optimum conditions as compared to the media before statistical optimization [52]. Three substrates, green peas, millet and ragi, were used in mixed SSF to produce statins in *A. terreus* MTCC 279. Various combinations of these substrates were designed by applying central composite design (CCD). Mixed substrates gave an 8.10-fold increase in compactin production in comparison to single substrate fermentation [97].

Statistical designing is an efficient approach that can significantly reduce experimental efforts that are not only required for optimisation studies but also includes scale-up and product development studies [102,103]. It also helps in confirmation of output response with least variability in comparison to conventional methods that are error prone and time consuming. The only drawback regarding the statistical approach includes lack of its knowledge and expertise among biologists and chemists to apply it in a multivariable natural system to achieve high levels with limited number of experimental trials. 

## 4. Conclusions

The microbial production of statins has provided an excellent therapy for hypercholesterolemia and led to the synthesis of novel statins by chemical synthesis. The use of *A. terreus* to achieve these outcomes is an excellent example of the exploitation of a microbe for useful purposes. Employing different kinds of optimization techniques and hyperproducers not only increase the yields but also results in economic production of these compounds.

## Figures and Tables

**Table 1:** Solid state fermentation (SSF) of *A. terreus* species using raw substrates.

*A. terreus* Strain	Solid Substrate	Yield	References
MTCC 279	Green peas, Millet, Ragi	389.34 mg/gds	[59]
ATCC 74135	Rice straw	0.261 mg/g	[60]
4	Wheat bran	9.7 mg/g	[61]
20	Oat bran	9.5 mg/g	[61]
PM3	Wheat bran	12.5 mg/g	[62]
UV 1718	Wheat bran	3.723 mg/g	[52]
*	Lactose, Soybean meal	19.95 mg/g	[63]
ATCC 20542	Rice powder, Glucose	2.9 mg/g	[64]

gds, g of dry support; * name not given.

**Table 2:** Carbon and nitrogen sources in submerged fermentation (SmF) of *A. terreus* species.

*A. terreus* Strain	Carbon Source	Nitrogen Source	Yield (mg/L)	References
ATCC 20542	Lactose, Glycerol	Yeast extract	161.8	[46]
Z15-7	Glycerol	Corn meal, Sodium nitrate	916.7	[57]
MUCL 38669	Lactose, Glucose	Peptonized milk, Yeast extract	212.5	[65]
LA414	Soluble starch	Yeast extract	952.7	[66]
LA414	Soluble starch	Sodium glutamate	523.9	[67]
LA414	Glycerol	Yeast extract	937.5	[68]
ATCC 20542	Lactose	Soybean meal	140	[69]
NRRL 255	Glucose, malt extract	Milk powder, Soybean meal	920	[50]
ATCC 20542	Lactose	Soybean meal	186.5	[23]
ATCC 20542	Lactose	Soybean meal	80	[70]
ATCC 20542	Lactose	Soybean meal	250	[51]
GD13	Lactose	Soybean meal	1242	[71]
*	Glucose	Soybean meal	110.78	[59]
ATCC 20542	Lactose	Yeast extract	83.8	[55]
*	Dextrose	Soy flour	100	[72]
20	Lactose	Yeast extract	120	[73]
ATCC 20542	Crude glycerol	Yeast extract	300	[74]

* Name not given.

**Table 3:** Effect of various additives on statin production by various *A. terreus* strains.

*A. terreus* Strain	Additive (Concentration)	Yield (mg/L)	Reference
ATCC 20542	Polyketide Antibiotics (50 mg/L)	952.7	[66]
ATCC 20542	Itaconic acid (0.5 g/L)	953.3	[47]
PM3	CMC (1%)	240	[62]
MUCL 38669	Linoleic acid (320 μM)	212.5	[65]
MUCL 38669	Butyrolactone I (100 nM)	3100	[78]
ATCC 20542	B-group vitamins (0.5–5 mg/L)	Unknown	[79]
ATCC 20542	Divalent metal cations (5 mM)	524	[67]

**Table 4:** Improved statin production through chemical and physical mutagenesis of *A. terreus*.

*A. terreus* Strain	Mode of Mutation	*A. terreus* Strain after Mutation	Improved Yield (mg/L)	Fold Increase	References
GD _13_	UV	EM _19_	1424	7.5×	[71]
20452	EMS	E354	60.3	4×	[82]
NRRL 265	UV	UV-4	977.1	3.5×	[84]
MTCC 10831	UV + EMS	SPUV002	663	1.8×	[83]
ATCC 20452	UV	LA414	883.2	3×	[85]
CA99	Heavy-ion beams	Z15-7	916.7	4×	[57]
AH6	UV	CB4	58	1.16×	[86]
20451	EMS+UV+NTG	DRCC 122	2200	1.73×	[75]
DRCC 86	EMS+UV	LS-3031	40	1.38×	[45]

**Table 5:** Statistical approaches for media optimisation for production of statins by *A. terreus.*

*A. terreus* Strain	Statistical Models	Statins	Yield	Reference
ATCC 20542	BBD	Lovastatin	186.5 mg/L	[23]
Strain not given	PB, CCD	Mevastatin	170.4 mg/L	[59]
ATCC 20542	PB, FD, RSM	Lovastatin	100 mg/L	[96]
MTCC 279	CCD	Compactin	389 mg/gds	[97]
MTCC 279	CCD	Lovastatin	1467 mg/gds	[97]
JX081272	Taguchi Design	Lovastatin	255 mg/L	[98]
UV 1718	RSM, CCD	Lovastatin	372 mg/g	[52]

RSM: Response surface methodology; CCD: Central composite design; BBD: Box−Behnken design; PB: Plackett–Burman; FD: factorial design; gds, g of dry support.

## Abbreviations

The following abbreviations are used in this manuscript:

gds: grams of dry support
SSF: solid state fermentation
CMC: carboxymethylcellulose
LDL: low density lipoprotein
EMS: ethyl methanesulfonate
NTG: *N*-Methyl-*N*’-nitro-*N*-nitrosoguanidine
